# Microvascular decompression for intermediate nerve neuralgia: a case report and literature review

**DOI:** 10.3389/fsurg.2024.1350075

**Published:** 2024-05-17

**Authors:** Kan Wang, Wenhao Li, Yujie Bo, Biao Li, Jinxin Wan, Luyan Mu, Yuwen Song

**Affiliations:** Department of Neurosurgery, The Fourth Affiliated Hospital of Harbin Medical University, Harbin, Heilongjiang, China

**Keywords:** intermediate nerve neuralgia, intermediate nerve, microvascular decompression, otalgia, functional neurosurgery

## Abstract

Intermediate nerve neuralgia (INN) is a rare craniofacial pain syndrome. The diagnosis of INN is challenging because of the complex ear sensory innervation that results in a clinical overlap with both trigeminal neuralgia (TN) and glossopharyngeal neuralgia (GPN). A 76-year-old woman with a remarkable medical history presented with right otalgia and mandibular pain for 7 years. Neurological examination revealed a diminished sensation in the distribution of the intermediate nerve (IN). Magnetic resonance imaging demonstrated an impression of the anterior inferior cerebellar artery (AICA) on the facial–vestibulocochlear nerve complex (VII/VIII complex). The patient underwent microvascular decompression (MVD) after long-term oral medication. We confirmed that the responsible vessel was close to the VII/VIII complex and isolated the vessel under the microscope via a right-sided suboccipital retrosigmoid approach. The patient's otalgia and mandibular pain disappeared after the operation. There were no additional neurological deficits. In conclusion, MVD is a safe and feasible option for patients with INN who fail to respond to adequate pharmacotherapy.

## 1 Introduction

Intermediate nerve neuralgia (INN), also called nervus intermedius neuralgia or geniculate neuralgia (GN), is a rare craniofacial pain syndrome that commonly causes severe unilateral pain in the external auditory canal (EAC) without any neurological deficits (1). The etiology of INN may be related to neurovascular conflict (NVC) or herpes zoster infection (2). The intermediate nerve (IN) contains three types of fibers: (1) general somatic afferent sensory fibers coming from the surface of the ear canal, tympanic membrane, retro-auricular skin, and regions within the pharynx; (2) special visceral afferent fibers coming from the anterior two-thirds of the tongue, floor of the mouth, and palate; and (3) general visceral efferent fibers innervating the lacrimal, sublingual, submandibular, and nasopalatine glands.

The IN exits the brainstem between the facial nerve and the vestibulocochlear nerve in the posterior cranial fossa before fusing with the facial nerve inside the internal acoustic meatus. Otalgia deep inside the ear can stem from the IN.

INN patients should begin with medical management, although the outcome is usually unsatisfactory, in which case, MVD or sectioning of the IN should be performed (3).

Here, we present a literature review and a case report to study the management of INN.

## 2 Case presentation

A woman in her early 60s with a remarkable medical history was admitted presenting with right otalgia and mandibular pain for 7 years. The right otalgia was located in the EAC, the auricle, and the deep part of the ear. The pain was described as a cutting-like sensation triggered by stimulation around the ear. It lasted from 10 s to several minutes and was occasionally accompanied by numbness at the base of the right tongue, which remitted spontaneously. The patient had a history of carbamazepine allergy and no history of ear herpes zoster infection. Prior to admission, she had undergone several acupuncture and physiotherapy sessions and had taken oral medications, such as mecobalamin and gabapentin, but with no significant improvement in symptoms. Physical examination did not show any unusual symptoms but the aforementioned pain. On admission, the patient had a Barrow Neurological Institute (BNI) pain intensity score of IV and a numerical rating scale (NRS) score of 9.

### 2.1 Examination

**Figure 1:**
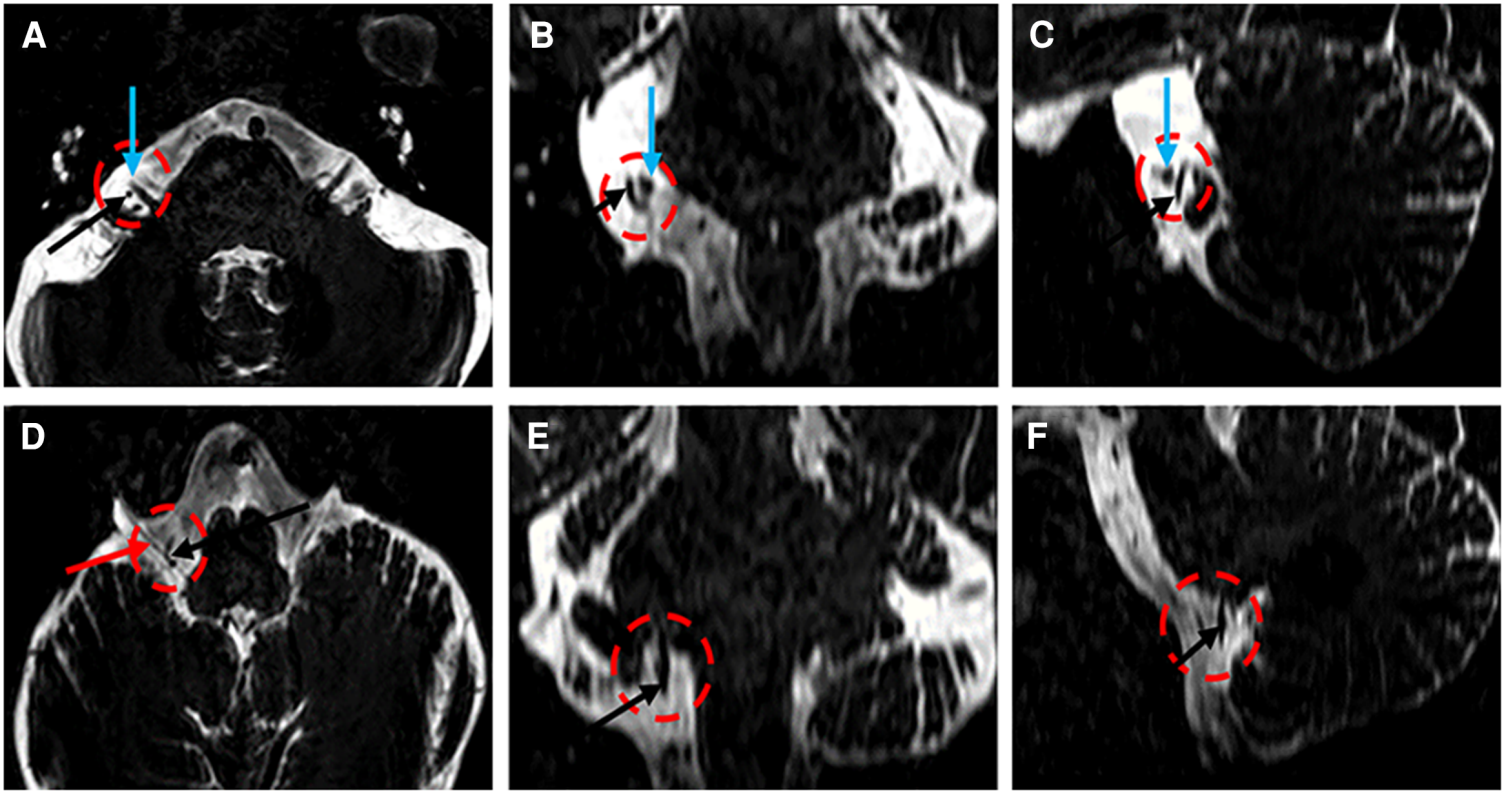
Three-dimensional T2-weighted images (**A–F**) demonstrating the AICA (**A–F**, black arrow), the VII/VIII complex (**A–C**, green arrow), and the vagus nerve (**D**, black arrow).

**Figure 2:**
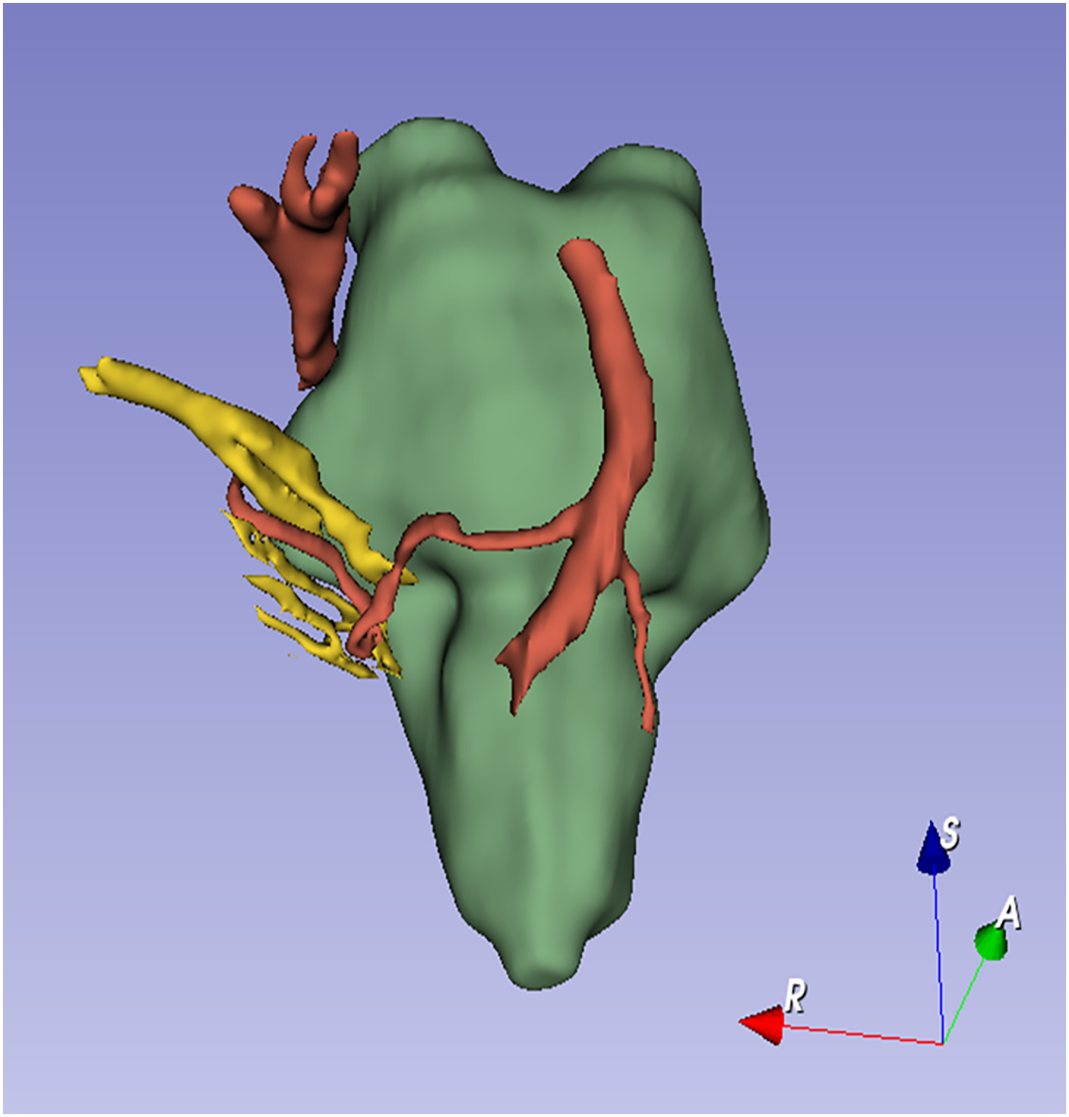
Three-dimensional reconstruction of MRI images according to the criteria for ICHD-3 intermediate neuralgia of the International Classification of Headache Disorders (4) (Table 1). The diagnosis of intermediate neuralgia was made based on the patient's history, imaging, and physical examination. Specifically, an operative picture that shows neurovascular compression on the nervus intermedius would help establish this diagnosis.

Magnetic resonance imaging (MRI) was performed to assess the neurovascular relationships in the cerebellopontine angle. Three-dimensional imaging, performed with 3D Slicer, clearly revealed a neurovascular contact between the facial–vestibulocochlear nerve complex (VII/VIII complex) and the anterior inferior cerebellar artery (AICA) (Figures 1A–C, 2). The AICA continued downward to make close contact with the vagus nerve (Figures 1D–F, 2).

**Table 1:** Summary of previous papers on surgical treatment for INN.

Author	Year	Number of case(s)	Treatment
Pulec et al. (1)	1976	15	Transection of the IN (4), GG (6), and IN and GG (5)
Rupa et al. (2)	1991	18	MVD (9) and transection of the IN (10), GG (10), ninth nerve (14), tenth nerve (11), tympanic nerve (4), and chorda tympani nerve (1)
Lovely et al. (3)	1997	14	MVD combined with transection of the IN (14)
Sachs. (4)	1968	4	Transection of the IN (4)
Tubbs et al. (5)	2013	1	MVD combined with transection of the IN (1)
Ozer et al. (6)	2009	1	MVD (1)
Zheng et al. (7)	2021	7	MVD (7)
Schroeder et al. (8)	2015	1	MVD (1)

GG, geniculate ganglion; TN, trigeminal neuralgia; MVD, microvascular decompression; IN, intermediate nerve.

According to the criteria for ICHD-3 intermediate neuralgia of the International Classification of Headache Disorders (4), the diagnosis of intermediate neuralgia was made based on the patient's history, imaging, and physical examination. Specifically, an operative picture that shows neurovascular compression on the nervus intermedius would help establish this diagnosis.

### 2.2 Surgical procedure

**Figure 3:**
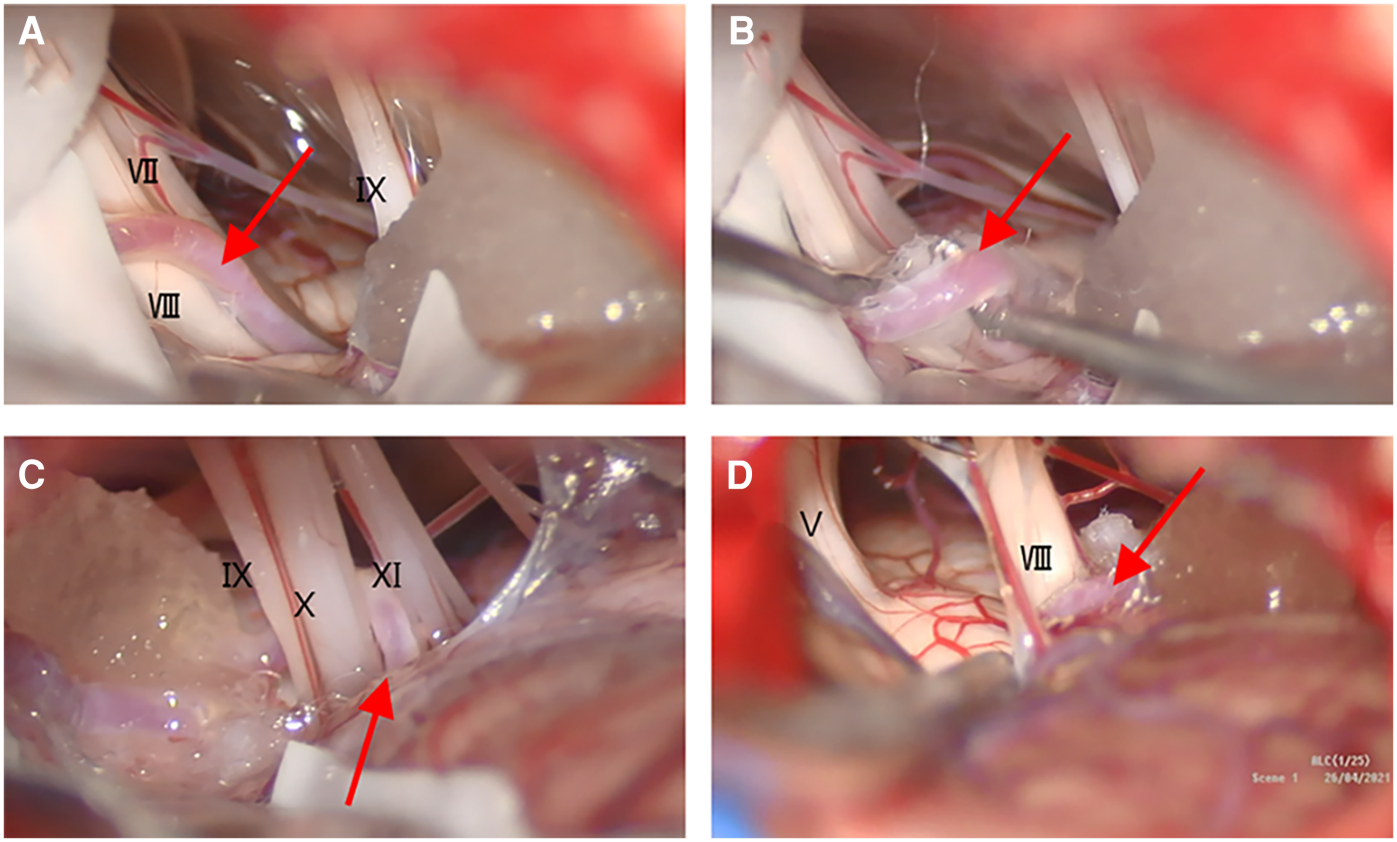
Intraoperative views under the microscope. (**A**) The ACIA (**A–D**, red arrow) had insinuated itself in the middle of the VII/VIII complex; (**B**) the AICA was lifted, and the Teflon felt was placed between the vessel and the VII/VIII complex; (**C**) the AICA near the caudal group of nerves; (**D**) no significant vascular compression of the trigeminal nerve root.

Microvascular decompression (MVD) in the right pontocerebellar horn region was performed under general anesthesia. The operation was performed via a right-sided suboccipital retrosigmoid approach. No obvious vascular compression was observed on the trigeminal nerve. The AICA between the facial and vestibulocochlear nerves compressed the cisternal segment and ventral side of the root entry zone (REZ). The offending artery was lifted, and a Teflon felt was placed between the arterial vessel and the VII/VIII complex. The exploration continued along the AICA toward the pedicle, and a vascular collateral was seen to form a close relationship with the cranial nerve of the caudal group. The Teflon felt was placed dorsally between the arterial vessel and the nerve of the caudal group (Figure 3).

### 2.3 Postoperative course

Postoperatively, the patient's otalgia and mandibular pain disappeared after MVD, and there were no additional neurological deficits. The BNI pain intensity score was 1, and the NRS score was 0. There was no recurrence within 6 months of follow-up.

Patient details were deidentified for this case report; hence, informed consent was not considered necessary, and ethical approval was not required for case report publication. We also obtained patient consent to treatment. The reporting of this study conforms to the CARE guidelines (5).

## 3 Discussion

INN is an uncommon cranial neuralgia syndrome. Its etiology remains unclear, although neurovascular compression of the IN is the most likely cause of this kind of otalgia. This otalgia is usually triggered by stimulation of the posterior wall of the EAC or periauricular region, which is usually caused by NVC with the IN by the AICA.

INN is difficult to diagnose directly; hence, diseases that are similar to INN should be eliminated first. These diseases include otitis externa and media and intracranial lesions in the cerebellopontine angle; malignancy of the pinna, EAC, temporal bone, or nasopharynx; temporomandibular joint diseases; lesions of dental origin; referred pain from nasopharyngeal and laryngeal lesions; vascular lesions; and rare syndromes, such as Eagle's syndrome (6). To eliminate all these causes of otalgia, the clinician should perform thorough neurological, dental, otological, and other ENT examinations.

To diagnose INN, differentiating it from other kinds of neuralgia, especially neuralgia supplied by cranial nerves V, VII, IX, and X, is essential. The two diseases most easily confused with INN are trigeminal neuralgia (TN) and glossopharyngeal neuralgia (GPN) because both are similar to INN in their pain characteristics. The location and distribution of the pain contribute to the differential diagnosis.

EAC pain can also occur with TN and GPN. Therefore, we need to gradually investigate the source of pain according to the diagnostic criteria (4). TN is the most common cause of facial pain. The typical location of the pain in TN is the second (maxillary) or third (mandibular) division of the trigeminal nerve. The right-side pain is usually mild, and the right side is also more likely to be affected than the left side (7). The most frequent trigger actions are touching the face, talking, chewing, and brushing teeth, while unusual trigger actions include flexing the trunk, contact with hot or cold food/water, speaking loudly, and turning the eyes to the right (8).

GPN is characterized by momentary, spasmodic, and acute stabbing pain in the posterior area of the throat, tonsillar fossa, base of the tongue, ear canal, and areas inferior to the angle of the mandible. GPN usually persists for several seconds and is triggered by swallowing, chewing, talking, coughing, or yawning. This kind of pain often overlaps with the pain of INN. Using local anesthesia to block local nerves in sequence may help identify the nervous origin (9).

To diagnose and assess patients with INN, 3.0 T MRI of the cerebellopontine angle is important. This may identify vascular compression of the nervus intermedius, although the positive rate is not 100%. In addition, vascular nerve compression cannot diagnose INN because many asymptomatic patients also have vascular nerve compression (10). However, the most important diagnostic criterion is an operative picture showing that neurovascular compression on the nervus intermedius is essential for the diagnosis of intermediate neuralgia.

Other investigations, such as auditory evoked brainstem responses, pure-tone audiometry, vestibular function tests, and computed tomography, can also help exclude other causes of otalgia.

All patients with INN should begin medicinal therapy, which is the first-line treatment for INN with varying success (11). Carbamazepine, lamotrigine, gabapentin, and tricyclic antidepressants, such as amitriptyline, are commonly given to patients with INN. Carbamazepine is the most commonly used medicine for INN and the most likely to achieve good clinical outcomes (12). The daily dose of carbamazepine ranges from 200 mg to 1,800 mg according to the patient's tolerability and sensitivity (13). The long-term use of carbamazepine will lead to low bone mineral density. Carbamazepine can also decrease the plasma concentration of warfarin and other drugs. Common side effects include, but are not limited to, tiredness, dizziness, ataxia, nausea, hyponatremia, and leucopenia (14).

Other medicines for the treatment of INN include oxcarbazepine (300–2,700 mg), lamotrigine (25–800,000 mg), gabapentin (300–3,600 mg), pregabalin (150–600 mg), baclofen (5–100 mg), phenytoin (50–500 mg), and botulinum toxin type A (25–195 units). Considering that these drugs have different sites of action, combinations of medicines for the treatment of INN may be better than single-drug therapy and can also treat patients who have single-drug resistance.

Regional nerve blockade through local anesthesia is an alternative to medical therapy. Many investigations suggest that local anesthesia is effective for all kinds of neuralgia, even beyond the period of conduction blockade, which means that all patients with ineffective drug treatment should receive regional nerve blockade before surgery.

When medicines and regional nerve blockade fail, surgery should be considered. There are many procedures for the treatment, but the most common forms of operation are MVD and intracranial sectioning of the nervus intermedius (15). We present herein a summary of previous papers on the surgical treatment of INN (Table 1). We found that with a deeper understanding of INN, the surgical treatment of INN gradually changed from transection of the IN to MVD combined with transection of the IN. The surgical methods selected in our case refer to the previous literature and verify and supplement them, which is believed to have a good reference value for subsequent INN patients (16–23).

Surgical treatment is an optional treatment for INN. There are two widely used options, namely, MVD of the IN at its REZ and transection of the nervus intermedius. MVD of the nervus intermedius is a surgery that relieves the vascular compression at its root entry area to the brainstem if there is a tortuous vascular loop. Usually, the compressing vessel is the AICA or the vertebral artery. This procedure aims to separate the nervus intermedius from the compressing vessel by inserting a piece of Teflon as MVD for TN. However, unlike the clinical efficacy of MVD for TN, there is still a lack of evidence to support the efficacy of MVD for intermediate neuralgia (24). Kenning et al. (25) performed and described MVD for intermediate neuralgia in detail.

Sectioning of the nervus intermedius is a surgery that directly cuts off the IN. This kind of operation can effectively relieve or even cure neuralgia. However, considering that the IN is composed of three kinds of fibers (26), transection of the IN may lead to a corresponding loss of nerve function. A number of case reports suggest that, even if transection of the IN relieves the pain, this surgery has some complications, including, but not limited to, cerebrospinal fluid leak, paresis of the ninth and tenth cranial nerves, facial paresis, facial numbness, chemical meningitis, and wound infection (27, 28). In addition, NVC may not be found by 3.0 T MRI. The patient's change from fear of the disease to understanding and relaxation now further proves the effectiveness of our treatment.

## 4 Conclusion

Considering that INN is similar to TN and GPN, the diagnosis must be made after these diseases are excluded. At present, special MRI sequences are an important choice for the diagnosis of the disease for their ability to find neurovascular compression of the complex of the seventh and eighth cranial nerves; however, this imaging manifestation must be combined with clinical manifestations to be meaningful because many asymptomatic patients also have this imaging manifestation. In addition, there are many patients with clinical manifestations without obvious imaging abnormalities, which makes the diagnosis of INN very difficult. More studies focusing on the etiology of INN are warranted.

## Data Availability

The original contributions presented in the study are included in the article/Supplementary Material, further inquiries can be directed to the corresponding author.
